# Supplementary material for: Marine cyanolichens from different littoral zones are associated with distinct bacterial communities
Source: PeerJ. 2018 Jul 17;6:e5208. doi: 10.7717/peerj.5208 (PMC6054067; doi:10.7717/peerj.5208)
Supplement: Supplemental Information 1 — Supplementary information comprising supplementary figures and details of the data analysis pipeline. Figure S1. Relative abundance (%) of bacterial and cyanobacterial sequences recovered from each lichen species. Xanthoria sp. Atl; from Brittany, and Xanthoria sp. Med. from the Mediterranean coast. Figure S2. Bray-Curtis dissimilarity dendrogram using the filtered dataset (retaining high variance OTUs) described in the main text. Figure S3. Comparison of relative abundance of core OTUs present in all replicates of two Xanthoria sp. sampled from the French Atlantic Ocean (Atl) and Mediterranean Sea (Med) coasts. OTUs are grouped at the family level when assigned or at the lowest taxonomic level assigned when family level was not available. Figure S4. Distribution of the major groups of Cyanobacteria associated with the different lichen species with the groups presented at the lowest assignable taxonomic level. [file peerj-06-5208-s001.docx]

**Supplementary Information**

**Supplementary Materials and Methods**

*Molecular identification of lichen species*

The morphological identification of the lichens was verified by sequencing the the ITS-5.8S rRNA region using the primers ITS1 (5’-TCCGTAGGTGAACCTTGCGG-3’) and ITS4 (5’-TCCTCCGCTTATTGATATGC-3’) (White et al., 1990) for the marine and maritime lichens (no clean sequences were obtained for the inland terrestrial lichens). For the inland terrestrial lichens, the genes for beta-tubulin and nuclear LSU rRNA were amplified using the primer pair Bt3-LM (5’-GAACGTCTACTTCAACGAG-3’) and Bt10-LM (5’-TCGGAAGCAGCCATCATGTTCTT-3’) for beta-tubulin (Myllys, Lohtander & Tehler, 2001) and the primer pair LR7 (5’- TACTACCACCAAGATCT-3’) (Vilgalys & Hester, 1990) and nrLSU0170 (5’-CCYTTCGACGACTCGAGT-3’) for the nuLSU rRNA gene.

Approximately 1-5 ng genomic DNA was amplified with the different primers sets (final concentration of primers at 0.4 µM) in reaction volumes of 10 µl using the KAPA2G Fast HotStart ReadyMix (Sigma-Aldrich, France) at 1X concentration. For amplification of the ITS region, the following cycling conditions were used: 95 °C 5 min followed by 30 cycles of 95 °C 15 s, 52 °C 15 s, 72 °C 15s followed by 72 °C 5 min. For the beta-tubulin and nuLSU genes, the following conditions were used: 95 °C 5 min followed by 37 cycles of 95 °C 15 s, 55 °C 15 s, 72 °C 15s followed by 72 °C 5 min. PCR products were checked by agarose gel electrophoresis and purified using the CleanSweep™ PCR Purification Reagent according to manufacturer’s instructions (ThermoFisher Scientific, France). Then approximately 10 ng of purified PCR products were sequenced using the BigDye™ Terminator v3.1 Cycle sequencing Kit (ThermoFisher Scientific, France) using the primer ITS1, Bt3-LM or LR7 (0.32 µM final concentration) in 10 µl reactions with 0.5 µl BigDye™ reaction mix and 1.75 µl 5X BigDye™ buffer. Cycle sequencing reactions were as follows: 40 cycles of 95 °C 10 s, 50 °C 5 s, 60 °C 2 min 30 s. Reactions were analysed with a 3130XL Genetic Analyzer (Applied Biosystems™, France) equipped with an 80 cm capillary array and base-called sequences were visualised and corrected using the software CodonCode Aligner (CodonCode Corporation, MA, USA). Corrected sequences were then analysed by blastn to find the closest relatives and the results are compiled in Table S2.

**16S rRNA gene data analysis pipeline**

*Sequence pre-processing (using USEARCH v8 unless QIIME or MOTHUR indicated)*

1. Merge pairs (0 mismatches in overlap region)

usearch8 -fastq_mergepairs 141003_SN1126_A_L001_HCI-2_R1.fastq -reverse 141003_SN1126_A_L001_HCI-2_R2.fastq -fastq_truncqual 3 -fastq_maxdiffs 0 -fastqout merged0.fastq

2. Quality filtering with usearch8 (maxee 1.0)

usearch8 -fastq_filter merged0.fastq -fastq_maxee 1.0 -fastaout reads0_filt.fasta -fasta_cols 0

3. Separate out eubacterial 16S reads in forward sense using the 341F primer (ligation on PCR was not directional so reads appear in both orientations)

grep -B1 "^.\{7\}CCTACGGG.GGC.GCAG" reads0_filt.fasta > eubfor0.fasta

4. Separate out eubacterial 16S reads in reverse sense using the 806R primer

grep -B1 "GACTAC..GGGTATCTAATCC" reads0_filt.fasta > eubrev0.fasta

5. Reverse complement reverse reads with QIIME command

adjust_seq_orientation.py -i eubrev0.fasta -r

6. Combine forward and reverse complemented reads in one file

cat eubrev0_rc.fasta eubfor0.fasta > euball0.fasta

7. Remove double hyphen that is introduced at beginning of sequence during the reverse complementing (otherwise the demultiplexing only assigns the forward reads)

sed s'/\--// g' euball0.fasta > eub0.fasta

8. Demultiplex reads using QIIME command to parse out reads to sample using the mapping file with no mismatches in barcode allowed

demultiplex_fasta.py -f eub0.fasta -m mapping_file_eub.txt -b 7 -e 0.5 --retain_unassigned_reads

9. Parse out reads to the different projects: MALICA and MOCK data set using QIIME command

extract_seqs_by_sample_id.py -i demultiplexed_seqs.fna -o malica.fasta -m mapping_file_eub.txt -s "Project:MALICA"

extract_seqs_by_sample_id.py -i demultiplexed_seqs.fna -o mock.fasta -m mapping_file_eub.txt -s "Project:MOCK"

10. Trim off primer sequences with MOTHUR allowing only exact matches to primers

File: 16Soligos.txt

forward CCTACGGGNGGCWGCAG

reverse GACTACHVGGGTATCTAATCC

trim.seqs(fasta=malica.fasta, oligos=16Soligos.txt)

trim.seqs(fasta=mock.fasta, oligos=16Soligos.txt)

11. Dereplicate sequences

usearch8 -derep_fulllength malica.trim.fasta -fastaout malicaunique.fasta -sizeout

12. Sort sequence abundance by size and discard singletons

usearch8 -sortbysize malicaunique.fasta -fastaout malicaunique2.fasta -minsize 2

*OTU clustering and modification of the OTU table*

13. Cluster sequences into OTUs at 97% using the UPARSE algorithm

usearch8 -cluster_otus malicaunique2.fasta -otus malicaotus.fa -uparseout malicauparseresults.txt -relabel OTU_ -sizein –sizeout

14. Chimera checking with unchime in *de novo* mode (optional as the majority of chimeras are removed during OTU clusterin)

usearch -uchime malicaotus.fa -uchimeout malicaresults2.uchime -uchimealns malicauchime2.alns -nonchimeras malicaotus_nochim.fa

15. Map OTUs back to the original filtered reads

usearch8 -usearch_global malica.trim.fasta -db malicaotus_nochim.fa -strand plus -id 0.97 -uc malicaotumap1.uc -threads 8

16. Make an OTU table using the Rober Edgar python script uc2otutab.py available from drive5.com that was modified by Mike Robeson (<https://groups.google.com/forum/#!msg/qiime-forum/zqmvpnZe26g/1F6LbQMkjToJ)> to uc2otutab_mod.py to allow extraction of OTU name before “_” instead of “;”. The script and the file must be in the same directory as the other scripts (uses modules die.py and Uc.py).

python uc2otutabmod.py malicaotumap1.uc > malicaotu_table1.txt

17. Remove cluster size from OTU name in OTU table

sed 's/;size.*;//g' malicaotu_table1.txt > malicaotu_table1mod.txt

18. Make a BIOM table

biom convert --table-type="otu table" -i malicaotu_table1mod.txt -o malica.biom

19. Remove cluster size from OTU representative sequence names in fasta file

sed 's/;size.*;//g' malicaotus_nochim.fa > malicaotus_nochim2.fa

20. Assign taxonomy using the RDP classifier and the GreenGenes 16S rRNA gene database (QIIME)

parallel_assign_taxonomy_rdp.py -c 0.6 --rdp_max_memory 4000 -O 8 -t /usr/local/data/gg_13_8_otus/taxonomy/97_otu_taxonomy.txt -r /usr/local/data/gg_13_8_otus/rep_set/97_otus.fasta -i /users/west/Illumina_raw_sequences/Merged_0/MALICA/malicaotus_nochim2.fa -o /users/west/Illumina_raw_sequences/Merged_0/MALICA/Assigned_taxonomy_rdp

21. Clean up taxonomy to remove unassigned levels

sed 's/;p__;c__;o__;f__;g__;s__ / /g' ./Assigned_taxonomy_rdp/malicaotus_nochim2_tax_assignments.txt | sed 's/;c__;o__;f__;g__;s__ / /g' | sed 's/;o__;f__;g__;s__ / /g' | sed 's/;f__;g__;s__ / /g' | sed 's/;g__;s__/ /g' | sed 's/;s__ / /g' > malicatax2.txt

22. Add taxonomy to BIOM table

biom add-metadata --sc-separated taxonomy --observation-header OTUID,taxonomy --observation-metadata-fp Assigned_taxonomy_rdp/malicatax2.txt -i malica.biom -o malicatax2.biom

23. Filter out Archaea, Chloroplast, Unclassified, Eukarya and mitochondria sequences (QIIME)

filter_taxa_from_otu_table.py -i malicatax2.biom -o malicatax2filt.biom -n c__Chloroplast,k__Archaea,k__Unclassified,f__mitochondria,k__Eukarya

24. Align sequences to reference with PYNAST (QIIME)

parallel_align_seqs_pynast.py -i ./malicaotus_nochim2.fa -t /usr/local/data/gg_13_8_otus/rep_set_aligned/97_otus.fasta -o ./pynast_aligned_seqs -O 8

25. Make a tree of sequences (QIIME)

Remove gaps

filter_alignment.py -i malicaotus_nochim2_aligned.fasta -s

Make the tree

make_phylogeny.py -i ./pynast_aligned_seqs/malicaotus_nochim2_aligned_pfiltered.fasta -o malica_repset.tree

26. Remove OTUs whose sequences did not align (QIIME)

filter_otus_from_otu_table.py -i malicatax2filt.biom -o malicatax2filt2.biom -e ./pynast_aligned_seqs/malicaotus_failures_ali.txt

27. Sort OTU table to order samples according to the mapping file (QIIME)

sort_otu_table.py -i malicatax2filt2.biom -o malicatax2filt2sort.biom -m mapping_file_malica2.txt -s SampleOrder

28. Filter the BIOM table to select non-cyanobacteria OTUs (QIIME)

filter_taxa_from_otu_table.py -i malicatax2filt2sort.biom -o malicatax2filt2sortnocyano.biom -n p__Cyanobacteria

29. Filter the BIOM table to select cyanobacteria OTUs (QIIME)

filter_taxa_from_otu_table.py -i malicatax2filt2sort.biom -o malicatax2filt2sortcyano.biom -p p__Cyanobacteria

**Supplementary Figures**

Figure S1. Relative abundance (%) of bacterial (bact) and cyanobacterial (cyano) sequences recovered from each lichen species.

Figure S2. Bray-Curtis dissimilarity dendrogram using the filtered dataset (retaining high variance OTUs) described in the main text. Lcr, *L. cristatum*; Lf, *L. fuscovirens*; La, *L. auriforme*; Sl, *S. lichenoides*; Xp, *X. parietina*; Xa, *X. aureola*; Lp, *L. pygmaea*; Lc, *L. confinis*.

Figure S3. Comparison of relative abundance of core OTUs present in all replicates of *Xanthoria* *aureola* and *Xanthoria parietina* sampled respectively from the French Atlantic Ocean and the Mediterranean Sea coasts. OTUs are grouped at the family level when assigned or at the lowest taxonomic level assigned when family level was not available.

Figure S4. Distribution of the major photobionts associated with the different lichen species with the groups presented at the lowest assignable taxonomic level. L.c, *L. confinis*; L.p, *L. pygmaea*;  SW, seawater ; L.a, *L. auriforme*; L.cr, *L. cristatum*; L.f, *L. fuscovirens*; S.l, *S. lichenoides*; Xa, *X. aureola*;  X.p, *X. parietina*.

**Supplementary References**

Myllys L., Lohtander K., Tehler A. 2001. β-Tubulin, ITS and Group I Intron Sequences Challenge the Species Pair Concept in Physcia aipolia and P. caesia. *Mycologia* 93:335–343. DOI: 10.2307/3761655.

Vilgalys R., Hester M. 1990. Rapid genetic identification and mapping of enzymatically amplified ribosomal DNA from several Cryptococcus species. *Journal of Bacteriology* 172:4238–4246.

White T., Bruns T., Lee S., Taylor J. 1990. Amplification and direct sequencing of fungal ribosomal RNA genes for phylogenetics. In: Innis M, Gelfand D, Shinsky J, White T eds. *PCR Protocols: A Guide to Methods and Applications*. Academic Press, 315–322.
