# Supplementary material for: Marine cyanolichens from different littoral zones are associated with distinct bacterial communities
Source: PeerJ. 2018 Jul 17;6:e5208. doi: 10.7717/peerj.5208 (PMC6054067; doi:10.7717/peerj.5208)
Supplement: Table S1 — List of lichens sampled with details of the different clusters, their geographical location and the temperature at the time of sampling. [file peerj-06-5208-s002.docx]

**Table S1. Sample location and environmental conditions**

| Lichen species | Cluster | Sample name | Longitude | Latitude | Temp air °C | Temp sea °C |
| --- | --- | --- | --- | --- | --- | --- |
| *Lichina confinis* | 1 | Lc1.1 | 48.629237 | -2.471686 | 18.5 |  |
|  |  | Lc1.2 |  |  |  |  |
|  |  | Lc1.3 |  |  |  |  |
|  | 2 | Lc2.1 | 48.629262 | -2.471969 |  |  |
|  |  | Lc2.2 |  |  |  |  |
|  |  | Lc2.3 |  |  |  |  |
|  | 3 | Lc3.1 | 48.629167 | -2.471954 |  |  |
|  |  | Lc3.2 |  |  |  |  |
|  |  | Lc3.3 |  |  |  |  |
| *Lichina pygmaea* | 1 | Lp1.1 | 48.62845 | -2.475342 |  | 18.5 |
|  |  | Lp1.2 |  |  |  |  |
|  |  | Lp1.3 |  |  |  |  |
|  | 2 | Lp2.1 | 48.628630 | -2.475420 |  |  |
|  |  | Lp2.2 |  |  |  |  |
|  |  | Lp2.3 |  |  |  |  |
|  | 3 | Lp3.1 | 48.628608 | -2.475486 |  |  |
|  |  | Lp3.2 |  |  |  |  |
|  |  | Lp3.3 |  |  |  |  |
| Seawater | 1 | SW1 | 48.62845 | -2.475342 |  | 18.5 |
|  |  | SW2 |  |  |  |  |
| *Xanthoria* *aureola*  (Atlantic) | 1 | Xa1.1 | 48.62967 | -2.47368 | 18.5 |  |
|  |  | Xa1.2 |  |  |  |  |
|  |  | Xa1.3 |  |  |  |  |
| *Xanthoria* parietina  (Mediterranean) | 1 | Xp1.1 | 42.49005 | 3.13049 | 12.5 |  |
|  |  | Xp1.2 |  |  |  |  |
|  |  | Xp1.3 |  |  |  |  |
| *Lathagrium auriforme* | 1 | La1.1 | 47.205637 | 15.399050 | 0 |  |
|  |  | La1.2 |  |  |  |  |
|  |  | La1.3 |  |  |  |  |
| *Lathagrium fuscovirens* | 1 | Lf1.1 | 47.205477 | 15.400819 | 3 |  |
| *Lathagrium cristatum* | 1 | Lcr1.1 | 47.205477 | 15.400819 |  |  |
|  |  | Lcr1.2 |  |  |  |  |
| *Scytinium lichenoides* | 1 | Sl.I.1.1 | 47.205249 | 15.398843 | 0 |  |
|  |  | Sl.I.1.2 |  |  |  |  |
|  | 2 | Sl.II1.1 | 47.205249 | 15.398843 |  |  |
|  |  | Sl.II1.2 |  |  |  |  |
|  |  | Sl.II1.3 |  |  |  |  |
