# Supplementary material for: Marine cyanolichens from different littoral zones are associated with distinct bacterial communities
Source: PeerJ. 2018 Jul 17;6:e5208. doi: 10.7717/peerj.5208 (PMC6054067; doi:10.7717/peerj.5208)
Supplement: Table S3 — List of sample IDs for each lichen sample and their corresponding barcode used to tag the forward primer 341F. [file peerj-06-5208-s004.docx]

**Table S3. Barcodes and primers for each sample**

| SampleID | Barcode | Linker Primer Sequence | Reverse Primer | Lichen Species |
| --- | --- | --- | --- | --- |
| Lc1.1 | CACACGT | CCTACGGGNGGCWGCAG | GACTACHVGGGTATCTAATCC | *L. confinis* |
| Lc1.2 | CACATTG | CCTACGGGNGGCWGCAG | GACTACHVGGGTATCTAATCC | *L. confinis* |
| Lc1.3 | CACCTAT | CCTACGGGNGGCWGCAG | GACTACHVGGGTATCTAATCC | *L. confinis* |
| Lc2.1 | CACTAAG | CCTACGGGNGGCWGCAG | GACTACHVGGGTATCTAATCC | *L. confinis* |
| Lc2.2 | CACTTCC | CCTACGGGNGGCWGCAG | GACTACHVGGGTATCTAATCC | *L. confinis* |
| Lc2.3 | CAGAGTT | CCTACGGGNGGCWGCAG | GACTACHVGGGTATCTAATCC | *L. confinis* |
| Lc3.1 | CAGGAGA | CCTACGGGNGGCWGCAG | GACTACHVGGGTATCTAATCC | *L. confinis* |
| Lc3.2 | CAGTCAT | CCTACGGGNGGCWGCAG | GACTACHVGGGTATCTAATCC | *L. confinis* |
| Lc3.3 | CATCGGT | CCTACGGGNGGCWGCAG | GACTACHVGGGTATCTAATCC | *L. confinis* |
| Lp1.1 | CATGCCT | CCTACGGGNGGCWGCAG | GACTACHVGGGTATCTAATCC | *L. pygmaea* |
| Lp1.2 | CATGTGG | CCTACGGGNGGCWGCAG | GACTACHVGGGTATCTAATCC | *L. pygmaea* |
| Lp1.3 | CATTCTG | CCTACGGGNGGCWGCAG | GACTACHVGGGTATCTAATCC | *L. pygmaea* |
| Lp2.1 | CCAACAT | CCTACGGGNGGCWGCAG | GACTACHVGGGTATCTAATCC | *L. pygmaea* |
| Lp2.2 | CCAAGTA | CCTACGGGNGGCWGCAG | GACTACHVGGGTATCTAATCC | *L. pygmaea* |
| Lp2.3 | CCAGTGT | CCTACGGGNGGCWGCAG | GACTACHVGGGTATCTAATCC | *L. pygmaea* |
| Lp3.1 | CCATACC | CCTACGGGNGGCWGCAG | GACTACHVGGGTATCTAATCC | *L. pygmaea* |
| Lp3.2 | CCGAATC | CCTACGGGNGGCWGCAG | GACTACHVGGGTATCTAATCC | *L. pygmaea* |
| Lp3.3 | CCGATAG | CCTACGGGNGGCWGCAG | GACTACHVGGGTATCTAATCC | *L. pygmaea* |
| SW1 | CCTCAAT | CCTACGGGNGGCWGCAG | GACTACHVGGGTATCTAATCC | None |
| SW2 | CCTGGAA | CCTACGGGNGGCWGCAG | GACTACHVGGGTATCTAATCC | None |
| La1.1 | CCTTAGA | CCTACGGGNGGCWGCAG | GACTACHVGGGTATCTAATCC | *L. auriforme* |
| La1.2 | CCTTGTC | CCTACGGGNGGCWGCAG | GACTACHVGGGTATCTAATCC | *L. auriforme* |
| La1.3 | CGAAGGT | CCTACGGGNGGCWGCAG | GACTACHVGGGTATCTAATCC | *L. auriforme* |
| Lf1.1 | CGCTGAT | CCTACGGGNGGCWGCAG | GACTACHVGGGTATCTAATCC | *L. fuscovirens* |
| Lcr1.1 | CGGTAGT | CCTACGGGNGGCWGCAG | GACTACHVGGGTATCTAATCC | *L. cristatum* |
| Lcr1.2 | CGTAACC | CCTACGGGNGGCWGCAG | GACTACHVGGGTATCTAATCC | *L. cristatum* |
| Sl.II1.1 | CGACACA | CCTACGGGNGGCWGCAG | GACTACHVGGGTATCTAATCC | *S. lichenoides* |
| Sl.II1.2 | CGAGCTA | CCTACGGGNGGCWGCAG | GACTACHVGGGTATCTAATCC | *S. lichenoides* |
| Sl.II1.3 | CGATTGC | CCTACGGGNGGCWGCAG | GACTACHVGGGTATCTAATCC | *S. lichenoides* |
| Sl.I.1.1 | CGCAATT | CCTACGGGNGGCWGCAG | GACTACHVGGGTATCTAATCC | *S. lichenoides* |
| Sl.I.1.2 | CGCATAA | CCTACGGGNGGCWGCAG | GACTACHVGGGTATCTAATCC | *S. lichenoides* |
| Xa1.1 | CCGCTTA | CCTACGGGNGGCWGCAG | GACTACHVGGGTATCTAATCC | *X. aureola* |
| Xa1.2 | CCGTTCT | CCTACGGGNGGCWGCAG | GACTACHVGGGTATCTAATCC | *X. aureola* |
| Xa1.3 | CCTATCA | CCTACGGGNGGCWGCAG | GACTACHVGGGTATCTAATCC | *X. aureola* |
| Xp1.1 | CGTACGA | CCTACGGGNGGCWGCAG | GACTACHVGGGTATCTAATCC | *X. parietina* |
| Xp1.2 | CGTCCTT | CCTACGGGNGGCWGCAG | GACTACHVGGGTATCTAATCC | *X. parietina* |
| Xp1.3 | CGTGTAC | CCTACGGGNGGCWGCAG | GACTACHVGGGTATCTAATCC | *X. parietina* |
| Mock | CTAACGG | CCTACGGGNGGCWGCAG | GACTACHVGGGTATCTAATCC | HM-782D even |
